# Serological survey on the prevalence of chicken infectious anemia virus in broiler breeder and layer farms in some selected areas of Bangladesh

**DOI:** 10.5455/javar.2021.h518

**Published:** 2021-06-25

**Authors:** Md. Al Arif Kabir, Sukumar Saha, Md. Golzar Hossain, Kamrul Ahmed Khan, Md. Alimul Islam, Lutfor Rahman

**Affiliations:** 1 Department of Microbiology and Hygiene, Bangladesh Agricultural University, Mymensingh, Bangladesh; 2 Poultry Care Lab, Paragon Group, Gazipur, Bangladesh

**Keywords:** Chicken infectious anemia, seroprevalence, ELISA, poultry, Bangladesh

## Abstract

**Objective:**

Chicken infectious anemia virus (CIAV) is an economically important emerging infection of poultry as it causes immunosuppression and reduces egg production. Although it is worldwide distributed and first reported (single case) in Bangladesh in 2002, no epidemiological and serological investigations have been conducted. The current study aimed to conduct a serological investigation on the prevalence of CIAV infection in broiler breeder and layer farms in some selected areas of Bangladesh.

**Materials and Methods:**

A total number of 460 sera samples were randomly collected from unvaccinated broiler breeder and layer flocks, of which 276 were from 11 broiler breeder farms and 184 from 12 layer farms. The sera samples were subjected to a commercially available enzyme-linked immunosorbent assay kit to observe antibodies induced by CIAV.

**Results:**

Results demonstrated that the overall prevalence of CIAV was 83.6% among a total of 460 samples. In broiler breeder birds, the prevalence was 89.9%, whereas it was 78.3% in layer birds. A higher number of female birds was found to be seropositive than male birds. However, chickens of all age groups were found to be susceptible to the virus.

**Conclusions:**

These results indicate the presence of CIAV in Bangladesh, which may be the sequel of naturally occurring either vertical or horizontal infection in all bird flocks tested without clinical symptoms of the disease. A further epidemiological investigation will be required, followed by molecular isolation and characterization of the virus for suitable vaccine candidate selection and/or preparation.

## Introduction

The poultry industry is one of the rapidly growing sectors in Bangladesh, and it plays a substantial role in poverty eradication and economic growth in emergent nations [1]. But nowadays, this industry is facing challenges posed by various pathogenic viral infections such as Newcastle disease virus, avian influenza virus, Marek’s disease virus (MDV), chicken infectious anemia virus (CIAV), infectious bursal disease virus (IBDV), avian reovirus infection, duck plague virus, etc. [2–6]. Among these diseases, chicken infectious anemia (CIA) is an important avian viral infection caused by the CIAV [7].

CIAV is a naked virus with a size of 25 nm, icosahedral in shape, whose DNA genome is circular and single-stranded, under the family Anelloviridae and genus *Gyrovirus* [8,9]. The chicken is believed to be a familiar host affected by CIAV without any age limitation for infection, although antibodies were observed in Japanese quail [10,11]. CIAV spreads via both direct exposure to infected chickens as a means of horizontal transmission and vertically from parents to offspring [12]. This virus causes aplastic anemia, hemorrhage in the skin, muscle, and other organs. Atrophy of the thymus and bone marrow with concomitant immunosuppression in 2–4 weeks-old chickens leads to an increased mortality and weight loss [13,14]. Due to immunosuppression caused by CIAV, opportunistic and/or secondary infection may occur [15]. Therefore, it is considered an important avian viral agent distributed worldwide [12]. CIAV antibodies can be detected from unvaccinated poultry flocks in many countries where enzyme-linked immunosorbent assay (ELISA) has been popularly used for seroprevalence studies [16–18]. ELISA can be used for rapid diagnosis with suitability, and a large number of samples can be tested at a time. The CIAV has been first recovered/identified from the bursa of Fabricius in 4-weeks-old birds from an acute infectious bursal disease outbreak in Bangladesh by using molecular technique [19]. To our knowledge, no report is available on CIAV status in Bangladesh. Here, a serological survey has been conducted to check the prevalence of anti-CIAV antibodies in CIAV-unvaccinated chickens of various layer and broiler breeder farms in some selected regions of Bangladesh.

## Materials and Methods

### Ethical approval

The serum samples have been collected by taking verbal consent from the farm owner. The ethical committee of the Bangladesh Agricultural University has approved the current study (No. AWEEC/BAU/2020(38)).

### Sources of samples

**Figure 1.**
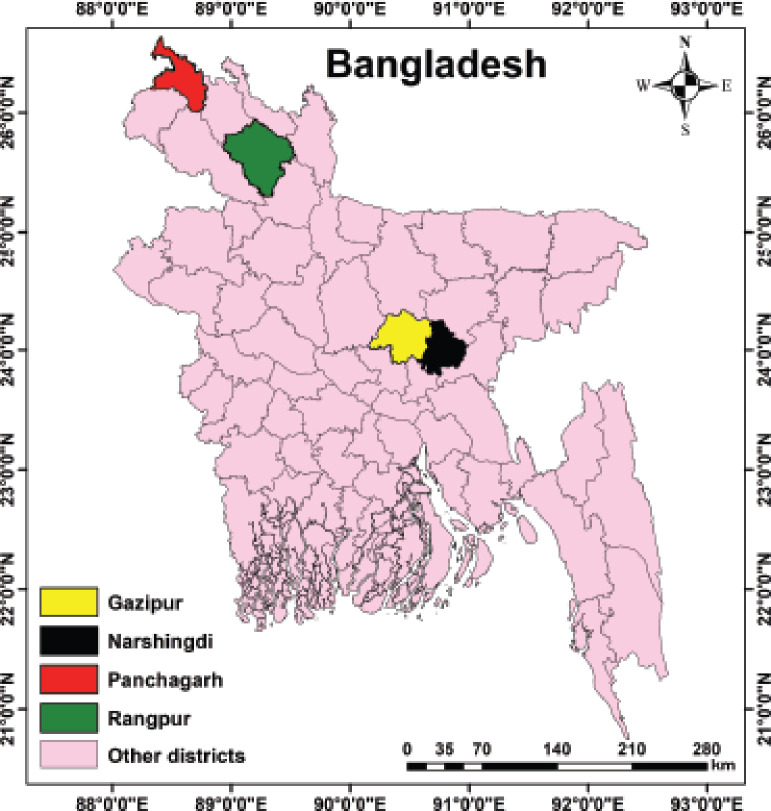
Sampling area map of selected districts of Bangladesh. Images were extracted from DIVA-GIS (http://www.diva-gis.org/) and provided by geographical information system. Finally, the map was created using ArcMap software (version 10.7).

A total number of 460 sera samples were randomly collected from CIAV-unvaccinated birds of different farms from four districts of Bangladesh, namely Gazipur (*n* = 253), Norshingdi (*n* = 33), Rangpur (*n* = 67), and Panchagarh (*n* = 107) (Fig. 1), of which, 276 samples (78 from male and 198 from female chickens) were from 11 broiler breeder (Cobb 500) farms and 184 (76 from male and 108 from female chickens) from 12 layers (BV 300) farms. The age of the chickens varied between 12 and 57 weeks. The sera samples were subjected to check the presence of antibodies against CIAV. The flocks were observed with no clinical signs or symptoms of CIAV.

### Preparation of serum samples

Wing veins of chickens were used to collect blood samples from the CIAV-unvaccinated flock using 3-ml-sized syringe maintaining aseptic conditions. Serum was prepared following the formerly mentioned method and kept at –20°C until further use [20,21].

### Enzyme-linked immunosorbent assay (ELISA) test

All the collected sera samples were subjected to indirect ELISA to observe serum antibodies against CIAV using a commercially available ELISA kit (X-OVO FLOCKSCREEN; Scotland, UK). The ELISA test was used following the manufacturer’s prescribed methods and manual for the analysis, interpretations, and correlations of data generated. A 1:500 dilution of serum was used. Optical density values were read at 550 nm using a SPECTRAmax^®^ ELISA reader (USA).

### Statistical analysis

The antibody titers are expressed as the arithmetical mean with standard deviation (SD) and coefficient of variation (CV). Prevalence and confidence intervals (Ci) were calculated using Microsoft Excel (version 2010) and Statistical Package for the Social Sciences (version 20). *p* < 0.05 is considered statistically significant. 

## Results

### Seroprevalence of CIAV according to areas

The serological test results depict an average of 83.6% (95% CI, 80.22%–86.98%) prevalence against CIAV in birds of the sampling areas. Chickens of the farms of Gazipur district had the highest prevalence of 87.0%, followed by Panchagarh (85.0%), Norshingdi (81.8%), and Rangpur (80.6%) (Table 1).

### Seroprevalence of CIAV farm-wise

The CIAV-specific antibodies have been detected in all flocks of broiler breeders, as revealed by ELISA. A total of 248 (89.9%) were found to be positive among 276 samples collected from 11 flocks. However, 28 (10.1%) samples were observed as negative. Three flocks (27.3%) were 100% positive, while eight other flocks from eight different farms were 95.2%, 80.0%, 84.0%, 83.3%, 81.8%, 85.7%, 96.3%, and 73.5% positive for specific CIAV antibodies. Among 11 farms, farm D showed the lowest CV as 10.9 (Table 2). In the case of layer flocks tested, out of 184 samples from a total of 12 flocks, 144 (78.3%) were found to be positive. At the flock level, only one flock (8.3%) was 100% positive, while 11 other flocks from 11 different farms were 76.5%, 82.1%, 76.7%, 84.6%, 81.0%, 81.0%, 61.5%, 90.9%, 66.7, 55.6%, and 50.0% positive for specific CIAV antibodies. Among all 12 farms, the lowest CV was recorded at 25.5 in farm J (Table 3).

**Table 1.** Demonstration of seroprevalence of CIAV by indirect ELISA in different experimental areas.

Sl. No.	District (Experimental areas)	Types of birds	No. of positive sera	Seroprevalence (%)	Overall Sero-prevalence according to district (%)	95% CI (Confidence interval)			Average of Sero-prevalence (%)	95% CI (Confidence interval)		
							L (%)	U (%)			L (%)	U (%)
1.	Gazipur	Broiler breeder	119	93.0	**87.0**	±4.14	82.86	91.14	**83.6**	±3.38	**80.22**	**86.98**
		Layer	101	80.8								
2.	Norshingdi	Broiler breeder	10	83.3	81.8	±13.16	68.64	94.96				
		Layer	17	81.0								
3.	Rangpur	Broiler breeder	36	83.7	80.6	±9.47	71.13	90.07				
		Layer	18	75.0								
4.	Panchagarh	Broiler breeder	83	89.2	85.0	±6.77	78.23	91.77				
		Layer	8	57.1								

L = Lower limit, U = Upper limit.

**Table 2.** Demonstration of seroprevalence of CIAV in broiler breeder flocks farm-wise.

District	Farm	No. positive/total tested samples	Sero-positive (%)	Antibody titer mean	Antibody titer (SD)	Antibody titer (CV)
Gazipur	A	80/84	95.2	3,225.5	1,272.0	39.4
	B	5/5	100.0	2,387.6	479.2	20.1
	C	4/5	80.0	2,145.1	479.2	22.3
	D	21/25	84.0	1,687.6	184.0	10.9
	E	9/9	100.0	1,600.0	423.3	26.5
Norshingdi	F	10/12	83.3	2,981.8	828.8	27.8
Rangpur	G	18/22	81.8	2,509.0	1,020.1	40.7
	H	18/21	85.7	2,922.1	1,115.3	38.2
Panchagarh	I	32/32	100.0	1,738.6	575.7	33.1
	J	26/27	96.3	1,537.3	218.2	14.2
	K	25/34	73.5	1,709.4	378.9	22.2

SD = Standard deviation, CV = Coefficient of variation.

### Seroprevalence of antibodies against CIAV according to the type of birds

in broiler breeder flocks, 248 (89.9%) samples out of 276 were seropositive, ranging from 86.35% to 93.45% at a 95% CI. The seroprevalences among broiler breeder birds in four districts were as follows: Gazipur (93.0%), Norshingdi (83.3%), Rangpur (83.7%), and Panchagarh (89.2%). In the case of the layer bird group, 144 (78.3%) samples out of 184 were found to be seropositive with a range of 72.34%–84.26% at 95% CI (Table 5). The seroprevalence in four districts was as follows: Gazipur (80.8%), Norshingdi (81.0%), Rangpur (75.0%), and Panchagarh (57.1%), as presented in Table 4.

### Seroprevalence of CIAV according to the sex of the birds

The results showed a higher number of female birds was found to be seropositive than male birds. Thus, in broiler breeder, out of 78 samples from male chickens, 64 (82.1%) samples were seropositive, and out of 198 samples from female chickens, 184 (92.9%) samples were seropositive. In the layer bird group, out of 76 samples from male chickens, 52 (68.4%) were seropositive. On the other hand, out of 108 samples from female chickens, 92 (85.2%) were found to be seropositive. In the overall seroprevalence analysis, out of 154 sera samples, 116 (75.3%) male chickens were CIAV positive with a range of 68.49%–82.11% at 95% CI, while out of 306 sera samples, 276 (90.2%) female sera samples were seropositive with a range of 86.87%–93.53% at 95% CI (Table 5).

**Table 3.** Demonstration of seroprevalence of CIAV in layer flocks farm-wise.

District	Farm	No. positive/total tested samples	Sero-positive (%)	Antibody titer mean	Antibody titer (SD)	Antibody titer (CV)
Gazipur	A	13/17	76.5	3,751.2	1,197.0	31.9
	B	5/5	100.0	2,147.8	624.1	29.1
	C	32/39	82.1	2,713.4	737.9	27.2
	D	23/30	76.7	2,048.3	569.5	27.8
	E	11/13	84.6	2,133.7	1,404.2	65.8
	F	17/21	81.0	2,471.7	976.9	39.5
Norshingdi	G	17/21	81.0	4,859.7	1,822.4	37.5
Rangpur	H	8/13	61.5	3,477.9	1,439.7	41.4
	I	10/11	90.9	2,851.6	1,117.6	39.2
Panchagarh	J	2/3	66.7	2,223.8	566.7	25.5
	K	5/9	55.6	3,244.4	3,125.0	96.3
	L	1/2	50.0	2,498.8	1,566.5	62.7

SD = Standard deviation, CV = Coefficient of variation.

**Table 4.** Demonstration of seroprevalence of CIAV according to type of birds.

District	Broiler breeder						Layer						*p* value	Odd ratio
	NP/TTS	Sero-prevalence (%)	OP (%)	95% CI			NP/TTS	Sero-prevalence (%)	OP (%)	95% CI				
					L (%)	U (%)					L (%)	U (%)		
Gazipur	119/128	93.0	**89.9**	±3.55	**86.35**	**93.45**	101/125	80.8	**78.3**	±5.96	**72.34**	**84.26**	**0.0005**	(1/0.406) = **2.46**
Norshingdi	10/12	83.3					17/21	81.0						
Rangpur	36/43	83.7					18/24	75.0						
Panchagarh	83/93	89.2					8/14	57.1						

*p*-value of overall prevalence (OP) calculated between broiler breeder and layer, odds ratio calculated between broiler breeder and layer.

NP = No. of positive, TTS = Total tested samples, OP = Overall prevalence, CI = Confidence interval, L = Lower limit, U = Upper limit.

### Seroprevalence of antibodies against CIAV according to the age of the birds

The sera samples were categorized into three groups based on the age of the birds (both broiler breeder and layer), such as 12–26 weeks, 27–41 weeks, and 42–57 weeks, which were examined for seroprevalence of CIAV. In the age group of 12–26 weeks, out of 30 samples subjected to the test, 26 (86.7%) were seropositive. In the 27–41 age group, out of 165 samples, 139 (84.2%) were seropositive. In the third category (42–57 age group), out of 265 samples, 227 (85.7%) were seropositive against CIAV. Among all age groups, the 12–26 age group showed the lowest CV at 42.4 (Table 6).

## Discussion

The current study focused on investigating the seroprevalence of CIAV in selected areas of Bangladesh and determining the extent of such prevalence regarding the breed of poultry (layer and broiler breeder), sex, and age. Hence, sera samples were collected from four selected areas, namely Gazipur, Norshingdi, Rangpur, and Panchagarh districts of Bangladesh, and indirect ELISA. The results of this test showed that CIAV is found in all bird flocks of selected areas of the country with a high prevalence rate. The results obtained in this study were correlated with the findings and prevalence of CIAV of many other parts of the world [22–25]. Recently, various reports from Asia and Africa have shown elevated seroprevalence in the respective poultry flocks, indicating CIAV to be an emerging virus worldwide [17,18,26–28]. Gazipur is a poultry hub in Bangladesh [29] where CIAV might have predisposed chickens through vertical and horizontal transmissions because CIAV is transmitted both horizontally and vertically [12]. This might be a plausible explanation for such a large number of sera samples being seropositive. However, such a finding correlated with that of Bhatt et al*.* [17], who carried out research work on the prevalence with 404 sera samples collected from 13 commercial layers located in four northern states of India and analyzed the existence of CIAV antibodies using an ELISA. Their screening revealed that CIAV antibodies were present in 86.88% of sera samples. A study carried out by Mcllroy might be cited where it was concluded that CIAV was a horizontally acquired infection and older chickens lacked maternal antibodies against CIAV [13]. Furthermore, it was also reported that the disease was caused by a virus surviving in the poultry environment and transmitted between flocks. The source of the virus either might be excreted by vertically infected flock mates or from the external introduction to the poultry farm.

**Table 5.** Demonstration of seroprevalence of CIAV according to sex.

Type of birds	Male						Female						*p* value	Odd ratio
	NP/ TTS	Sero-prevalence (%)	OP (%)	95% CI			NP/TTS	Sero-prevalence (%)	OP(%)	95% CI				
					L (%)	U (%)					L (%)	U (%)		
Broiler breeder	64/78	82.1	**75.3**	±6.81	**68.49**	**82.11**	184/198	92.9	**90.2**	±3.33	**86.87**	**93.53**	**0.000**	**(1/0.3318) = 3.014**
Layer	52/76	68.4					92/108	85.2						

*p*-value of overall prevalence (OP) calculated between male and female, odds ratio calculated between females and males.

NP = No. of positive, TTS = Total tested samples, OP = Overall prevalence, CI = Confidence interval, L = Lower limit, U = Upper limit.

**Table 6.** Demonstration of seroprevalence of CIAV according to the age of the birds.

Age group (weeks)	No of positive /total tested samples	Seroprevalence (%)	Antibody titer mean	Antibody titer (SD)	Antibody titer (CV)
12–26	26/30	86.7	2,170.0	920.3	42.4
27–41	139/165	84.2	2,554.0	1,225.6	48.0
42–57	227/265	85.7	2,660.6	1,352.1	50.8

SD = Standard deviation, CV = Coefficient of variation.

The CIAV is mainly controlled by a maternal antibody derived from the breeder flock either by vaccination or natural infection [30]. The symptomatic disease occurs when birds get an infection at 2 weeks of age, but this infection may be prevented if the offspring get enough maternal antibodies from the breeder hens [12]. Maternal antibodies are highly effective and completely prevent the clinical disease caused by CIAV by 2–3 weeks of age [30,31]. Chicks can become age-resistant to CIAV when the antibody disappears.

The study was also concerned with seroprevalence investigation according to types of birds and found a higher rate of seroprevalence in both broiler breeder and layer chickens. Our results are also supported by the observation of previous researchers who also reported a high prevalence of CIAV antibodies in commercial poultry flocks [17,18]. It may be noted that CIAV prevalence was predominant in broiler breeder birds than layers of birds (*p *< 0.05). Such an odds ratio implied that the risk of being infected by CIAV of a broiler breeder bird group is 2.46 (1/0.406) times more than that of a layer bird group. Regarding the relationship of CIAV infection with the sex of birds, it was found that female chickens had higher seropositivity than their male counterparts (*p *< 0.05). The odds ratio implied that the risk of being infected by CIAV of a female bird group is 3.014 (1/0.3318) times more than that of a male bird group. In response to this study, a similar piece of research work carried out by Goryo et al. [32] might be mentioned where 20.9% and 2.4% mortality rates were reported in male and female chickens, respectively, in Japanese poultry flocks. In this context, it may be plausible that the birds, which have higher immunity, are usually less susceptible to a particular infection, and mortality rates would be lower. So, the clinical outbreak of the CIAV has not been reported so far in Bangladesh. Of note, the actual cause of difference of CIAV-antibody level between male birds and female is yet unknown and needs detailed investigation.

The other aspect of this study was to determine the magnitude of prevalence of CIAV in respect of age of birds, and the observations of Canal et al. [12] and Sharma et al. [18] also supported our analysis on age-related seroprevalence in broiler breeder and layer flocks. They detected a high level of antibodies against CIAV in broiler breeder flocks at the age of 6–55 weeks and in layer flocks at the age of 52–69 weeks. The first serological investigation conducted in Bangladesh revealed that the bird flocks carry CIAV infection with a high prevalence rate. It might be due to introducing this virus into the poultry flocks and spreading continuously throughout the country as the virus is highly contagious. The appearance of antibodies in the birds denotes natural infections because it is known that the vaccination program to control the CIAV is not practiced in Bangladesh [7]. CIAV outbreaks in the flocks are correlated with the absence of anti-CIAV antibodies in the corresponding parent flocks [33,34]. The CIAV infection in the chicks at the first stage of life, e.g., rearing period, manifesting clinical disease can be avoided if enough maternal antibodies are transferred to the offspring from their parent stocks. This is again supported by Yuasa et al. [35] and Yuasa et al. [36], as they mentioned that immunocompetent chicks develop resistance to CIAV at 4 weeks of age, while immunosuppression caused by coinfection with CIAV and either MDV or IBDV harms maternal immunity. Therefore, it could be logical to vaccinate parent stock against CIAV along with vaccinations against IBDV and MDV to keep away vertical transmission of the CIAV and to protect the offspring by maternal antibodies of CIAV.

## Conclusion

The CIAV appeared to be the highest in broiler breeder in comparison with layer birds. The female chickens had higher seropositivity than male chickens, although all ages of birds, irrespective of broiler breeder and layer, are susceptible to that virus. Nevertheless, it should be forgotten that the number of sera samples was not equal in all events of parameters used in the experiment. This remained a weakness in carrying out the study of seroprevalence, which is to be considered while undertaking such investigation in the future. Therefore, detailed investigation on the CIAV and its molecular epidemiology and characterization of the virus need to be conducted in the future.

## List of abbreviations

CIAV: Chicken infectious anemia virus; CIA: Chicken infectious anemia; ELISA: Enzyme-linked immunosorbent assay; MDV: Marek’s disease virus; IBDV: infectious bursal disease virus; SD: Standard deviation; CV: Coefficient of variation; SPSS: Statistical Product and Service Solutions; CI: Confidence interval.

## References

[1] Hamid MA, Rahman MA, Ahmed S, Hossain KM (2017). Status of poultry industry in Bangladesh and the role of private sector for its development. Asian J Poult Sci.

[2] Rimi NA, Hassan MZ, Chowdhury S, Rahman M, Sultana R, Biswas PK (2019). A decade of avian influenza in Bangladesh: where are we now?. Trop Med Infect Dis.

[3] Rahman MA, Rahman MM, Abdullah MS, Sayeed MA, Rashid MH, Mahmud R (2019). Epidemiological assessment of clinical poultry cases through the government veterinary hospital-based passive surveillance system in Bangladesh: a case study. Trop Anim Health Prod.

[4] Islam MS, Sabuj AAM, Haque ZF, Pondit A, Hossain MG, Saha S (2020). Seroprevalence and risk factors of avian reovirus in backyard chickens in different areas of Mymensingh district in Bangladesh. J Adv Vet Anim Res.

[5] Hossain MG, Azad MTA, Akter S, Amin MM, Saha S (2014). Biological characterization and determination of comparative efficacy of an inactivated Newcastle disease virus vaccine prepared from velogenic strain. Afr J Microbiol Res.

[6] Khan KA, Islam MA, Sabuj AAM, Bashar MA, Islam MS, Hossain MG (2021). Molecular characterization of duck plague virus from selected Haor areas of Bangladesh. Open Vet J.

[7] Ali MZ, Dahiya SS, Moula MM, Kumar S (2019). Efficacy of chicken anemia vaccine in broiler parent stock. Bangladesh J Vet Med.

[8] Rosario K, Breitbart M, Harrach B, Segales J, Delwart E, Biagini P (2017). Revisiting the taxonomy of the family Circoviridae: establishment of the genus Cyclovirus and removal of the genus Gyrovirus. Arch Virol.

[9] Li Y, Fang L, Cui S, Fu J, Li X, Zhang H (2017). Genomic characterization of recent chicken anemia virus isolates in China. Front Microbiol.

[10] Erfan AM, Selim AA, Naguib MM (2018). Characterization of full genome sequences of chicken anemia viruses circulating in Egypt reveals distinct genetic diversity and evidence of recombination. Virus Res.

[11] Al-Ajeeli K, Al-Rubayee H, Alazawy A (2018). Sero-prevalence of chicken anemia virus in local fowls and Japanese quails. Indian J Nat Sci.

[12] Canal CW, Ferreira DJ, Macagnan M, Fallavena LC, Moraes HL, Wald Vb (2004). Prevalence of antibodies against chicken anaemia virus (CAV) in broiler breeders in Southern Brazil. Pesqui Vet Bras.

[13] McIlroy SG, McNulty MS, Bruce DW, Smyth JA, Goodall EA, Alcorn MJ (1992). Economic effects of clinical chicken anemia agent infection on profitable broiler production. Avian Dis.

[14] Todd D (2000). Circoviruses: immunosuppressive threats to avian species: a review. Avian Pathol.

[15] Gimeno IM, Schat KA (2018). Virus-induced immunosuppression in chickens. Avian Dis.

[16] Vagnozzi AE, Espinosa R, Cheng S, Brinson D, O’Kane P, Wilson J (2018). Study of dynamic of chicken infectious anaemia virus infection: which sample is more reliable for viral detection?. Avian Pathol.

[17] Bhatt P, Shukla S, Mahendran M, Dhama K, Mm C, Kataria J (2011). Prevalence of chicken infectious anaemia virus (CIAV) in commercial poultry flocks of Northern India: a serological survey. Transbound Emerg Dis.

[18] Sharma R, Tiwari K, Chikweto A, Thomas D, Stratton G, Bhaiyat M (2014). Serologic evidence of chicken infectious anemia in layer and broiler chickens in Grenada, West Indies. Vet World.

[19] Islam MR, Johne R, Raue R, Todd D, Muller H (2002). Sequence analysis of the full-length cloned DNA of a chicken anaemia virus (CAV) strain from Bangladesh: evidence for genetic grouping of CAV strains based on the deduced VP1 amino acid sequences. J Vet Med B Infect Dis Vet Public Health.

[20] Hossain MG, Saha S, Akter S, Islam MA, Amin MM (2017). Molecular detection, biological characterization and evaluation of protective potentiality of a velogenic strain of Newcastle disease virus isolate of Bangladesh. Biotechnol J Int.

[21] Sabrin MS, Saha S, Amin MM, Hossain MG (2012). Immune response and protective potential following vaccination against Newcastle disease virus and fowl cholera in naked neck chickens. Bangladesh Vet.

[22] Yao S, Tuo T, Gao X, Han C, Yan N, Liu A (2019). Molecular epidemiology of chicken anaemia virus in sick chickens in China from 2014 to 2015. PLoS One.

[23] Mahzounieh M, Salehi T (2005). Serologic evidence of chicken infectious anemia in commercial chicken flocks in Shahrekord, Iran. Int J Poult Sci.

[24] Ou SC, Lin HL, Liu PC, Huang HJ, Lee MS, Lien YY (2018). Epidemiology and molecular characterization of chicken anaemia virus from commercial and native chickens in Taiwan. Transbound Emerg Dis.

[25] Shettima Y, ElYuguda A, Oluwayelu D, Abubakar M, Hamisu T, Zanna M (2017). Seroprevalence of chicken infectious anemia virus infection among some poultry species in Maiduguri, Nigeria. J Adv Vet Anim Res.

[26] Hadimli HH, Erganiş O, Güler L, Uçan US (2008). Investigation of chicken infectious anemia virus infection by PCR and ELISA in chicken flocks. Turk J Vet Anim Sci.

[27] Hegazy A, Abdallah F, Abd-El L, Samie L, Nazim AA (2010). Chicken infectious anemia virus (CIAV) in broilers and laying hens in Sharkia Province, Egypt. J Am Sci.

[28] Snoeck CJ, Komoyo GF, Mbee BP, Nakouné E, Le Faou A, Okwen MP (2012). Epidemiology of chicken anemia virus in Central African Republic and Cameroon. Virol J.

[29] Khaleda S (2013). The poultry value chain and sustainable development of poultry microenterprises that utilize homestead lands: a case study in Gazipur, Bangladesh. Land Use Policy.

[30] Fatoba AJ, Adeleke MA (2019). Chicken anemia virus: a deadly pathogen of poultry. Acta Virol.

[31] Otaki Y, Saito K, Tajima M, Nomura Y (1992). Persistence of maternal antibody to chicken anaemia agent and its effect on the susceptibility of young chickens. Avian Pathol.

[32] Goryo M, Suwa T, Matsumoto S, Umemura T, Itakura C (1987). Serial propagation and purification of chicken anaemia agent in MDCC-MSB1 cell line. Avian Pathol.

[33] Yuasa N, Imai K, Watanabe K, Saito F, Abe M, Komi K (1987). Aetiological examination of an outbreak of haemorrhagic syndrome in a broiler flock in Japan. Avian Pathol.

[34] Vielitz E, Landgraf H (1988). Anaemia-dermatitis of broilers: field observations on its occurrence, transmission and prevention. Avian Pathol.

[35] Yuasa N, Taniguchi T, Yoshida I (1979). Isolation and some characteristics of an agent inducing anemia in chicks. Avian Dis.

[36] Yuasa N, Imai K (1986). Pathogenicity and antigenicity of eleven isolates of chicken anaemia agent (CAA). Avian Pathol.

